# CT-based synthetic contrast-enhanced dual-energy CT generation using conditional denoising diffusion probabilistic model

**DOI:** 10.1088/1361-6560/ad67a1

**Published:** 2024-08-02

**Authors:** Yuan Gao, Richard L J Qiu, Huiqiao Xie, Chih-Wei Chang, Tonghe Wang, Beth Ghavidel, Justin Roper, Jun Zhou, Xiaofeng Yang

**Affiliations:** 1 Department of Radiation Oncology and Winship Cancer Institute, Emory University, Atlanta, GA, United States of America; 2 Department of Medical Physics, Memorial Sloan Kettering Cancer Center, New York, NY, United States of America

**Keywords:** diffusion probabilistic model, single-energy CT, contrast-enhanced, dual-energy CT, deep learning

## Abstract

*Objective.* The study aimed to generate synthetic contrast-enhanced Dual-energy CT (CE-DECT) images from non-contrast single-energy CT (SECT) scans, addressing the limitations posed by the scarcity of DECT scanners and the health risks associated with iodinated contrast agents, particularly for high-risk patients. *Approach.* A conditional denoising diffusion probabilistic model (C-DDPM) was utilized to create synthetic images. Imaging data were collected from 130 head-and-neck (HN) cancer patients who had undergone both non-contrast SECT and CE-DECT scans. *Main Results.* The performance of the C-DDPM was evaluated using Mean Absolute Error (MAE), Structural Similarity Index (SSIM), and Peak Signal-to-Noise Ratio (PSNR). The results showed MAE values of 27.37±3.35 Hounsfield Units (HU) for high-energy CT (H-CT) and 24.57±3.35HU for low-energy CT (L-CT), SSIM values of 0.74±0.22 for H-CT and 0.78±0.22 for L-CT, and PSNR values of 18.51±4.55 decibels (dB) for H-CT and 18.91±4.55 dB for L-CT. *Significance.* The study demonstrates the efficacy of the deep learning model in producing high-quality synthetic CE-DECT images, which significantly benefits radiation therapy planning. This approach provides a valuable alternative imaging solution for facilities lacking DECT scanners and for patients who are unsuitable for iodine contrast imaging, thereby enhancing the reach and effectiveness of advanced imaging in cancer treatment planning.

## 1. Introduction

Dual-energy computed tomography (DECT), first conceived in 1976 (Rutherford *et al* 1976), represents an evolution in CT technology, offering enhanced spectral tissue characterization that surpasses the capabilities of conventional single-energy CT (SECT) scans. This advanced imaging technique utilizes two distinct x-ray energy levels, enabling material decomposition by capturing and differentiating the unique attenuation properties of mediums at low and high x-ray energies (McCollough *et al* 2020). Utilizing this technique, the advantages of DECT over SECT have been well established. These include enhanced proton range uncertainty and mass density mapping (Taasti *et al* 2017, Chang *et al* 2023), the ability to generate iodine maps for enhanced lesion characterization (Kim *et al* 2013, Mileto *et al* 2015), and overall improved image quality (Yue *et al* 2018, Yang *et al* 2023).

Contrast-enhanced DECT (CE-DECT) is notably invaluable among the advanced clinical applications of DECT, extending beyond its traditional roles in diagnostic radiology and radiation oncology to various medical fields, including cardiology, pulmonology, urology, and vascular surgery, among others. In cardiology, despite advancements in prevention and treatment, coronary artery disease (CAD) remains the leading cause of death (Danad *et al* 2015). CE-DECT shows great potential in assessing CAD, as it can distinguish and quantify plaque composition within the arteries and evaluate myocardial perfusion, thereby improving the diagnosis and management of ischemic heart disease. In pulmonary medicine, CE-DECT is particularly effective for evaluating lung parenchyma and pulmonary vasculature. Its capability to provide detailed images of blood flow and lung perfusion makes it especially valuable in detecting and characterizing pulmonary embolism (Pontana *et al* 2008, Grob *et al* 2019). CE-DECT significantly enhances the visualization of vascular structures, playing a crucial role in vascular surgery for planning and evaluating endovascular procedures. It is instrumental in assessing aortic aneurysms and vascular stents and is also used extensively in preoperative planning for vascular surgeries (Agostini *et al* 2019, Hamid *et al* 2021).

However, the high costs of DECT-capable scanners limit their accessibility across imaging centers (Sodickson *et al* 2021). In addition to scanner availability, DECT scans may not be suitable for certain patients, such as those with increased body habitus (Patel *et al* 2017). Furthermore, iodine contrast agent injections are not suitable for patients with allergies to iodine or those with kidney diseases (Jean-Marc *et al* 2005, Davenport *et al* 2020). These constraints impede the universal adoption of the CE-DECT, thus limiting its potential benefits for a broader patient demographic.

A potential solution could be applying artificial intelligence (AI) to generate synthetic CE-DECT images, circumventing the scenarios where DECT scanners are unavailable and offering an alternative for patients who might otherwise be ineligible due to factors like increased body habitus or iodinated contrast allergies. Recent research has focused on training AI to transform images from various modalities, including but not limited to SECT, Cone-beam CT (CBCT), and Magnetic resonance imaging (MRI), into synthetic DECT images. This transformation is achieved through image-to-image translation using deep learning (DL) networks, as demonstrated in the following studies (Bredfeldt *et al* 2017, Lei *et al* 2019, Harms *et al* 2020, Liu *et al* 2021, Chang *et al* 2022, Gao *et al* 2022, Jeong *et al* 2023). The aim of current image-to-image translation methodologies is to infer DECT image applications that can aid radiation oncologists in developing treatment plans. However, to date, there is limited literature on the specific topic of synthesizing CE-DECT from non-contrast SECT images. Previous research has mainly centered on generating synthetic non-contrast DECT (Kawahara *et al* 2021, Jeong *et al* 2023) or CE-SECT (Kim *et al* 2021, Choi *et al* 2021b, Chun *et al* 2022) from non-contrast SECT images. To our knowledge, the synthesis of CE-DECT has not been previously attempted.

In this study, we introduced a framework based on a conditional denoising diffusion probabilistic model (DDPM) (Ho *et al* 2020) framework for CE-DECT generation based on non-contrast SECT. The diffusion model, an emerging image generative approach, has garnered significant attention in the field of medical imaging (Pan *et al* 2023) due to its superior performance over GAN-based models in image synthesis tasks (Dhariwal and Nichol 2021). Diffusion model, particularly DDPM has been first used in under-sampled MRI reconstruction (Peng *et al* 2022, Xie and Li 2022b), and Since then, DDPM has been applied to various synthesis tasks in medical imaging (Xie and Li 2022a). GANs, including variants like CycleGAN and Wasserstein CycleGAN, are powerful for image-to-image translation tasks but pose significant challenges in implementation and training. Issues such as mode collapse (Lee *et al* 2018), hyperparameter sensitivity, and training instability can lead to unrealistic image generation and require careful management of various parameters. In contrast, DDPMs are analytically principled, easier to train, and produce high-quality images (Croitoru *et al* 2022, Kazerouni *et al* 2022). Additionally, although CycleGAN is beneficial for unpaired image-to-image translation tasks, it is not ideally suited for this project’s requirements, where we possess ground truth images enabling us to perform supervised learning. For our DL model training, we employed paired datasets consisting of CE-DECT, including both high-energy and low-energy CT images, alongside non-contrast SECT images. In this setup, the CE-DECT images were treated as the target distribution, while the non-contrast SECT images served as the conditioning variable. The trained model is adept at progressively converting standard Gaussian noise into the desired CE high-energy CT (H-CT) and CE low-energy CT (L-CT) images.

To the best of our knowledge, this is the first study to implement a conditional DDPM for the synthesis of iodine map images using non-contrast SECT images, specifically targeting head-and-neck (HN) cancer patients. The proposed method, using DL techniques, can accurately create synthetic CE-DECT images from non-contrast SECT scans. This is beneficial for imaging centers lacking DECT scanners and for patients who are unsuitable for iodine injections. Furthermore, this technique could also extend its benefits to patients in specialties like cardiology, pulmonology, and urology.

## 2. Materials and methods

In the study, we developed a framework to generate synthetic CE-DECT images from non-contrast SECT, organized into two phases: training and application. We utilized data from 130 patients, strictly dividing it into a training dataset with randomly chosen 120 patients and a test dataset with the remaining 10 patients. To evaluate the effectiveness of our novel method, we chose Pixel-to-Pixel GAN (Pix2PixGAN) and convolutional neural network (CNN) as benchmark reference models. All three models were trained on 2D transverse slices in the sagittal direction. The evaluation process comprehensively incorporates both quantitative and visual assessments. Some key metrics were utilized to provide a detailed understanding of the models’ performance.

### 2.1. Image acquisition and data preprocessing

In this study, we analyzed radiotherapy simulation images from 130 head and neck (HN) cancer patients treated at our institute. These patients were prescribed by radiation oncologists to undergo CE-DECT scans immediately following their non-contrast SECT scans, with the time interval between the two scans ranging from 7 to 20 min, depending on the day’s clinical process efficiency. During the CT simulations, patients’ HN areas were immobilized using thermoplastic masks, and they were guided by radiotherapy therapists to remain still on the CT couch. The CT images were acquired using a Twin-Beam DECT scanner (SOMATOM Definition Edge, SIEMENS Healthineers, Forchheim, Germany). The non-contrast SECT scans were performed at 120 kVp with a FLAT filter and auto exposure control, while the CE-DECT scans utilized a twin-filter of gold (Au) for low energy and tin (Sn) for high energy, facilitating x-ray spectrum separation. In our workflow, the non-contrast SECT images were registered to the CE-DECT images using Velocity (Varian Medical Systems, Palo Alto, CA) via a rigid plus deformable registration process. These deformed non-contrast SECT images were the primary input for our proposed methodology, while the CE-DECT including high and low energy (H-CT & L-CT) serves as the ground truth.

### 2.2. DDPM

**Figure 1.**
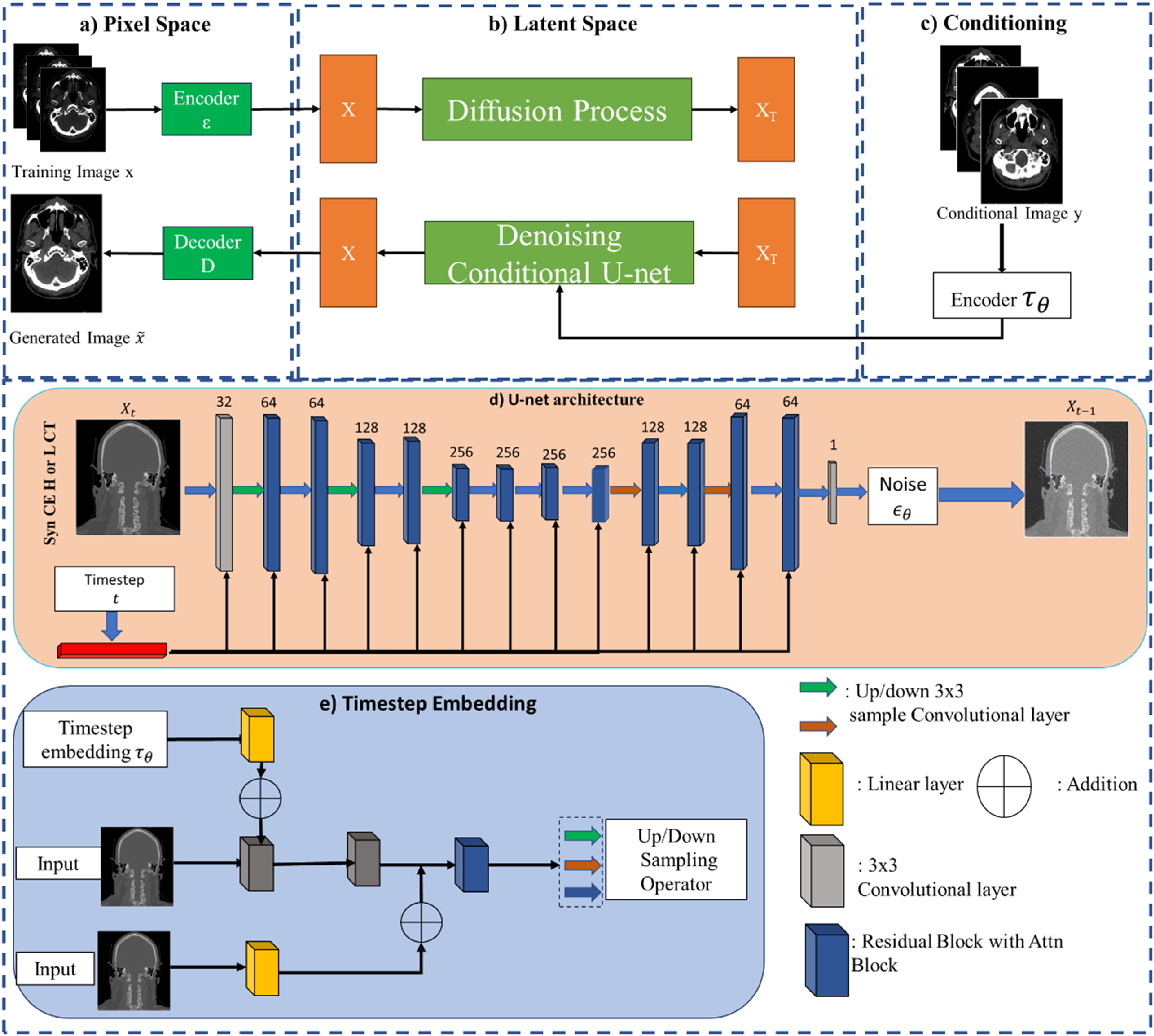
(a) Pixel Space: This section includes the encoder (ϵ) and decoder (D). The input image is the ground truth H-CT/L-CT, and the output image is the synthetic H-CT/L-CT generated by the model. (b) Latent Space: This section illustrates the diffusion process (forward process) and the reverse diffusion process. It shows how the noisy image X_t_ transitions through these processes. (c) Conditional Input: This section shows the non-contrast SECT image used as the conditional input for the model. (d) U-net Architecture: This section provides the detailed structure of the U-net used in this work. The architecture includes multiple convolutional layers with varying dimensions, up sampling and down sampling operations, and skip connections that help in preserving image details during denoising. (e) Timestep Embedding: This section shows the embedding of the timestep t, integrated into the U-net architecture. The embedding helps the model understand the current stage of the diffusion process, guiding the denoising more effectively.

The DDPM is a specific type of diffusion model, which belongs to a class of latent variable models. It employs a Markov chain process to transform a standard Gaussian distribution into the target data distribution (Ho *et al* 2020). This parameterization in DDPM effectively enables the gradual conversion of simple distributions into complex data distributions through a series of learned steps. Assuming the target data ${x_0}$ follows a distribution $p\left( {{x_0}} \right)$, a sequence of progressively corrupted images ${x_1},\,{x_2},\, \cdots ,\,{x_T}$ can be created through a series of forward diffusion processes, as illustrated by the green-arrow flow in figure 1(a). These processes are Markovian in nature, where each step progressively adds noise to the data, leading to a sequence where the images become increasingly corrupted. The formulation of this process is detailed in equations (1) and (2). where $T$ represents the total number of diffusion steps, ${\beta _t} \in \left( {0,1} \right)$ is a hyper-parameter controlling the incremental Gaussian noise, and $\mathcal{N}\left( {{x_t};\mu ,\sigma } \right)$ represents a Gaussian distribution of mean $\mu $ and variance $\sigma $. During the diffusion process, at each step $t$, noise is added according to the specified ${\beta _t}$, gradually transforming the data ${x_0}$ into a more noise-corrupted version ${x_t}$, until it ${x_T}$ reaches after $T$ steps

\begin{align*}P\left( {{x_t}{\text{|}}{x_{t - 1}}} \right) &amp; = \mathcal{N}\left( {{x_t};\sqrt {1 - {\beta _t}} {x_{t - 1}},{\beta _t}{\boldsymbol{I}}} \right)\end{align*}

\begin{align*}P\left( {{x_{1:T}}{\text{|}}{x_0}} \right) &amp; = \mathop {\mathop \prod \nolimits }\limits_{t = 1}^T P\left( {{x_t}{\text{|}}{x_{t - 1}}} \right).\end{align*}

Given the properties of the Gaussian distribution, ${x_t}$ at any arbitrary step can be directly computed from the initial image ${x_0}$. Consequently, equation (2) can be written into equation (3) with ${\alpha _t}: = 1 - {\beta _t}$ and ${\bar \alpha _t}: = \mathop \prod \nolimits_{i = 1}^t {\alpha _i}$: This reformulation leverages the fact that the Gaussian noise added at each step is independent and identically distributed, allowing for a direct calculation of ${x_t}$ from ${x_0}$ without needing to sequentially apply each diffusion step. This approach simplifies computation and provides a more efficient way to model the diffusion process

\begin{align*}p\left( {{x_t}{\text{|}}{x_0}} \right) &amp; = \mathcal{N}\left( {{x_t};\sqrt {{{\bar \alpha }_t}} {x_0},\left( {1 - {{\bar \alpha }_t}} \right)\boldsymbol{I}} \right).\end{align*}

In practical implementation, the forward process of the diffusion model can be simplified using the reparameterization trick. This approach allows for more efficient computation, particularly in the training stage. The reparameterization trick involves expressing the random variable ${x_t}$ as a deterministic function of the initial data ${x_0}$ and an independent noise variable. The process can be formulated as shown in equation (4), where the one-step calculation of ${x_t}$ is directly derived from ${x_0}$ and a noise term. This method reduces the computational complexity of the model and is particularly beneficial during training, where efficient and repeated calculations of ${x_t}$ are required. The reparameterization trick thus enables a more straightforward and effective way to train the diffusion model

\begin{align*}{x_t} &amp; = \sqrt {{{\bar \alpha }_t}} {x_0} + \sqrt {1 - {{\bar \alpha }_t}} \epsilon \end{align*}

where $\epsilon \sim N\left( {0,1} \right)$, as the number of steps $T$ approaches infinity, ${x_T}$ converges to isotropic Gaussian noise. By applying Bayes’ theorem, the posterior at each step in the reverse process is also modeled as a Gaussian distribution, conditioned on both ${x_T}$ and ${x_0}$. This reverse process can be expressed as shown in equation (5)

\begin{align*}p\left( {{x_{t - 1}}{\text{|}}{x_t},{x_0}} \right) &amp; = \mathcal{N}\left( {{x_{t - 1}};{{\tilde \mu }_t}\left( {{x_t},{x_0}} \right),{{\tilde \beta }_t}{\boldsymbol{I}}} \right).\end{align*}

In this equation, ${\tilde \mu _t}\left( {{x_t},{x_0}} \right)$ and ${\beta _t}$ are parameters of the Gaussian distribution at each step of the reverse process, and their expressions are provided in equation (6). These parameters are crucial for effectively modeling the reverse diffusion process, where the model gradually denoises the data, transforming the noisy distribution back to the original data distribution. This reverse process is central to the operation of the DDPM, enabling it to generate high-quality samples from a distribution that closely approximates the target data distribution

\begin{align*}{\text{with}}\;{\tilde \mu _t}\left( {{x_t},{x_0}} \right) &amp; = \frac{{\sqrt {{{\bar \alpha }_{t - 1}}} {\beta _t}}}{{1 - {{\bar \alpha }_t}}}{x_0} + \frac{{\sqrt {{{\bar \alpha }_t}} \left( {1 - {{\bar \alpha }_{t - 1}}} \right)}}{{1 - {{\bar \alpha }_t}}}{x_t}\;{\text{and}}\;{\tilde \beta _t} = \frac{{1 - {{\bar \alpha }_{t - 1}}}}{{1 - {{\bar \alpha }_t}}}{\beta _t}.\end{align*}

From an isotropic Gaussian distribution $p\left( {{x_T}} \right)$ with a sufficiently large $T$, it is possible to iteratively generate a sample in the target distribution $p\left( {{x_0}} \right)$ using the posterior distribution $p\left( {{x_{t - 1}}{\text{|}}{x_t}} \right)$. While ${\tilde \beta _t}$ can be computed directly with the know parameters ${\bar \alpha _t}$and ${\beta _t}$, ${\tilde \mu _t}\left( {{x_t},{x_0}} \right)$ is incomputable due to the unknown distribution of ${x_0}$. In our implementation, the DDPM framework approximates ${\tilde \mu _t}\left( {{x_t},{x_0}} \right)$ from ${x_t}$ using a neural network parameterized by $\theta $ and $t$, as shown in equation (7). This enables the network to predict the mean value of the reverse process distribution at each step. Consequently, equation (5), which describes the reverse diffusion process, can be approximated by equation (8). This approach allows for the practical application of DDPM in generating samples from the target distribution, overcoming the challenge of not knowing the exact distribution of ${x_0}$

\begin{align*}{\tilde \mu _t}\left( {{x_t},{x_0}} \right) &amp; \approx {\mu _{\theta ,t}}\left( {{x_t}} \right)\end{align*}

\begin{align*}p\left( {{x_{t - 1}}{\text{|}}{x_t},{x_0}} \right) &amp; \approx \mathcal{N}\left( {{x_{t - 1}};{\mu _{\theta ,t}}\left( {{x_t}} \right),{{\tilde \beta }_t}{\boldsymbol{I}}} \right).\end{align*}

The loss function can be written as equation (9), where ${\epsilon _{\theta ,t}}\left( {{x_t}} \right) \approx \epsilon $

\begin{align*}{\text{Loss}} &amp; = ||\epsilon - {\epsilon _{\theta ,t}}\left( {{x_t},t} \right)||_2^2.\end{align*}

The original DDPM, primarily designed for general image creation, is not optimally suited for generating images with specific contextual alignment. This limitation is particularly relevant in our study, which focuses on conditional image-to-image translation. It is crucial to highlight that in our study, the proposed DDPM was trained separately for generating H-CT and L-CT images from corresponding non-contrast SECT pairs. This separate training approach is crucial because, in our application, the synthetic L-CT and H-CT images must directly and independently correspond to the input non-contrast SECT images. This independent training ensures that the model effectively learns the unique characteristics and relationships between the SECT images and their respective H-CT or L-CT counterparts, leading to more accurate and contextually aligned synthetic images. This requirement is distinct from merely producing a random sample within the broader target distributions of H-CT or L-CT. Our approach, therefore, modifies the original DDPM framework to ensure precise alignment between the input SECT images and the corresponding synthetic L-CT and H-CT outputs. To control sample generation in diffusion models, several methods have been proposed to enforce specific conditions (Choi *et al* 2021a, Peng *et al* 2022, Saharia *et al* 2022, Xie and Li 2022b) in this specific project, the input SECT image y is concatenated with sample ${x_t}$ along the channel dimension to serve as a static guidance in each sampling step (Saharia *et al* 2022). This integration of the condition $y$ into the reverse process alters the noise estimator in the DDPM from ${\epsilon _{\theta ,t}}\left( {{x_t}} \right)$ to ${\epsilon _{\theta ,t}}\left( {{x_t},y} \right)$, and the posterior distribution becomes ${q_\theta }\left( {{x_{t - 1}}{\text{|}}{x_t},y} \right)$.Given a corresponding H-CT/L-CT and SECT pair $\left( {x,y} \right)$, the noise-prediction loss function (equation (9)) can be written as equation (10). This modification enables the model to generate more contextually aligned images by incorporating the conditioning information from the SECT image directly into the diffusion process. This approach is particularly effective for tasks where the generated image must maintain a specific relationship with an input image, as is the case in our study of synthesizing L-CT and H-CT images from non-contrast SECT images

\begin{align*}{\text{Loss}} &amp; = ||\epsilon - {\epsilon _{\theta ,t}}\left( {{x_t},t,y} \right)||_2^2.\end{align*}

With a network trained to approximate $\epsilon $ at each diffusion step, it becomes possible to invert the diffusion process. This inversion allows for the reconstruction of an original image residing in the distribution $p\left( {{x_0}} \right)$ from a Gaussian noise ${x_T}$, a process depicted by the yellow flow in figure 1(a). The reverse Markovian processes, which enable this reconstruction, are detailed in equations (11) and (12). In these equations, the trained network uses the estimated $\epsilon $ values to iteratively denoise the image, starting from the Gaussian noise ${x_T}$ and progressively reconstructing the data back to its original form in ${x_0}$. This reverse process is a crucial aspect of the diffusion model, allowing it to generate high-quality images that accurately reflect the characteristics of the target distribution

\begin{align*}{q_\theta }\left( {{x_{t - 1}}{\text{|}}{x_t},y} \right) &amp; = \mathcal{N}\left( {{x_{t - 1}};\frac{1}{{\sqrt {{\alpha _t}} }}\left( {{x_t} - \frac{{{\beta _t}}}{{\sqrt {1 - {{\bar \alpha }_t}} }}{\epsilon _{\theta ,t}}\left( {{x_t},y} \right)} \right),{{\tilde \beta }_t}{\boldsymbol{I}}} \right)\end{align*}

\begin{align*}{q_\theta }\left( {{x_{0:T}}{\text{|}}y} \right) &amp; = q\left( {{x_T}} \right)\mathop {\mathop \prod \nolimits }\limits_{t = 1}^T {q_\theta }\left( {{x_{t - 1}}{\text{|}}{x_t},y} \right).\end{align*}

The specifics of the training and sampling procedures for the proposed conditional DDPM (C-DDPM) algorithm are outlined in previous work (Peng *et al* 2023). In this approach, instead of training $T$ completely distinct networks to predict ${\epsilon _{\theta ,t}}\left( {{x_t},y} \right)$ at each diffusion step, a single, unified noise-prediction model with time-embedding, as introduced by Ho *et al* (2020), was utilized across all $T$ steps. This model leverages a U-net architecture enhanced with Shifted-window attention modules (Swin) and residual blocks to predict the noise at each time step. The detailed architecture of the U-net structure is depicted in figure 1(b), which illustrates how the various components of the U-net are configured and interact with each other. Additionally, the workflow for time-embedding, which is a crucial aspect of the model allowing it to adapt its predictions based on the specific diffusion time step, is shown in figure 1(c). This integration of time-embedding into the U-net structure with Swin and residual blocks facilitates a more precise and efficient noise prediction, enhancing the overall performance of the C-DDPM in generating high-quality synthetic images.

### 2.3. Reference models

Two reference models were implemented in this work: a Pix2PixGAN and a CNN. We chose these two models for the following reasons. GAN-based models are well-known for their excellent performance in image synthesis tasks, making them a strong benchmark. However, GAN-based models are often challenging to optimize. Therefore, we also compared our method with a CNN to highlight the performance differences and optimization challenges between traditional convolutional networks and GAN-based models.

#### 2.3.1. Pix2PixGAN

The Pix2PixGAN, also known as a conditional GAN, is a versatile tool designed for general-purpose image-to-image translation. It requires co-registered images with pixel-wise correspondence for its training, making it suitable for tasks where paired data is available. This model was originally developed by Isola *et al* (2017). In our study, we utilized a 2D Pix2PixGAN to generate synthetic CE-DECT. The structural details of the Pix2PixGAN network used in our project are elaborated in our previous publication (Gao *et al* 2023b). This includes information about the architecture of both the generator and the discriminator components of the Pix2PixGAN. Additionally, the specific formulations of the generator and discriminator loss functions, which are critical for the training and performance of the Pix2PixGAN in our application, are also detailed in the same work. These loss functions play a pivotal role in guiding the network to generate high-quality and contextually accurate synthetic iodine maps, crucial for our study’s objectives. Furthermore, the generator and discriminator modules have been specifically optimized for this task, ensuring tailored performance enhancement.

#### 2.3.2. CNN

CNN incorporating residual blocks, as introduced by He *et al* (2016), have shown remarkable results in image-to-image translation tasks, especially when the source and target images exhibit substantial similarity. This similarity is closely mirrored in the relationship between CE-DECT and non-contrast SECT images. The intricacies of employing CNNs with residual blocks for this specific application are elaborated in our previous work (Gao *et al* 2023a). Residual blocks, while maintaining the size of the feature map, significantly contribute to network optimization. They allow for deeper networks by alleviating the vanishing gradient problem and facilitate the learning of identity functions, ensuring that the addition of layers does not degrade the network’s performance. This feature is particularly beneficial in tasks like image-to-image translation, where preserving and accurately transforming intricate details from the source to the target image is crucial. The residual blocks, therefore, play a vital role in enhancing the overall efficacy and accuracy of the CNN in such applications.

### 2.4. Implementation and evaluation

The non-contrast SECT and H-CT/L-CT images were processed as 256 × 256 2D slices. With each of the 120 patients contributing 200 slices, the training dataset comprised a total of 24 000 slices. The original clinical images were 512 × 512 pixels; however, due to GPU memory constraints (24 GB), processing larger 512 × 512 slices were not feasible as it required 15 GB of memory for 256 × 256 slices (see table 1). Given these limitations, we opted for 256 × 256 slices. This study focuses on vascular tissue in the head, specifically excluding any blank CT slices. Consequently, a set of 200 axial slices per patient was deemed appropriate for our analysis. For the proposed C-DDPM, we set the total number of diffusion time steps, $T$, to 1000, with a noise variance schedule linearly increasing from ${\beta _1} = {10^{ - 4}}$ to ${\beta _T} = 0.02$. The Adam optimizer was selected with a learning rate of $2 \times {10^{ - 4}}$, betas of 0.9 and 0.999, and eps of 10^−8^. We maintained a batch size of 1 throughout the experiments. All the experiments were conducted using PyTorch 1.12 on a 24 GB Nvidia RTX 4090 GPU (operating system is Windows 10 Pro). The training computation cost comparison is listed in table 1.

**Table 1.** Computation cost between propose and reference methods.

	GPU memory (GB)	CPU memory (GB)	Parameter number	Training time (s/epoch)	Training epochs	Inference time (s/slice)
DDPM (Proposed method)	14.9	5	83 211 521	690	1000	85
CNN	12.5	5.3	21 841 410	500	650	<1
Pix2PixGAN	11.9	5.2	21 841 410 (Generator)	140	750	<1
			3057 377 (Discriminator)			

For quantitative evaluations in our study, synthetic CE-DECT images were compared with their ground truth counterparts using three key metrics: mean absolute error (MAE), peak signal-to-noise ratio (PSNR), and structural similarity index measure (SSIM). It is crucial to note that this comparison was conducted across three different anatomical planes: sagittal, coronal, and axial views of the synthetic CE-DECT images. The test data was prepared in these three views as 2D slices. However, it is important to highlight that the DL models used for generating these synthetic images were trained exclusively on sagittal view slices. This methodology presents an interesting aspect of model generalizability and the capability of the trained models to perform effectively on views they were not explicitly trained on. Evaluating the synthetic images in all three anatomical planes provides a more comprehensive understanding of the model’s performance and its applicability in clinical settings, where multi-planar image analysis is crucial for accurate diagnosis and treatment planning.

MAE measures the magnitude of the difference between the generated image and the ground truth image, as shown in equation (13). Where $f\left( {i,j} \right)$ is the value of pixel $\left( {i,j} \right)$ in the ground truth image, $t\left( {i,j} \right)$ is the value of pixel $\left( {i,j} \right)$ in generated image, and ${n_x}{n_y}$ are the number of voxels, ${{\text{n}}_{\text{z}}}$ is the number of slices. The MAE provides a straightforward measure of how closely the generated image matches the ground truth on a pixel-by-pixel basis, making it a valuable tool for assessing the accuracy of image synthesis models in applications,

\begin{align*}{\text{MAE}} &amp; = \frac{1}{{{n_x}{n_y}{n_z}}}\sum\limits_{i,j,k}^{{n_x}{n_y}{n_z}} \left| {f\left( {i,j} \right) - t\left( {i,j} \right)} \right|.\end{align*}

PSNR is an image quality metric that defines the ratio between the maximum possible power of a signal and the power of corrupting noise affecting the fidelity of its representation. PSNR is calculated with equation (14). Where MAX is the maximum signal intensity possible and MSE is the mean-squared error of the image. This equation essentially expresses the PSNR in decibels (dB). A higher PSNR value typically indicates a higher quality of reconstruction or generation, as it suggests a lower level of error or noise in the generated image compared to the original. PSNR is particularly useful in scenarios like image compression or image synthesis, where it is important to quantify the loss of quality due to the process. In medical imaging, where accuracy and clarity are paramount, PSNR can be a critical metric for evaluating the performance of image generation models like those used for creating synthetic CT images,

\begin{equation*}{\text{PSNR}} = 10 \times {\log _{10}}\left( {\frac{{{\text{MA}}{{\text{X}}^{\text{2}}}}}{{{\text{MSE}}}}} \right).\end{equation*}

SSIM is a metric used to measure the similarity between two images. It provides a more accurate and perceptual assessment of image quality compared to MAE and PSNR, which do not align well with human visual perception (Wang *et al* 2004). The measures between two images *x* and *y* of common size *N* $ \times $ *N* is shown in equation (15). SSIM is especially useful for evaluating image quality in ways that align more closely with the way humans perceive images. SSIM values range between −1 and 1, where 1 indicates perfect similarity. This measure is particularly valuable in fields like medical imaging, where the structural integrity and perceptual quality of an image are crucial for accurate diagnosis and analysis.

\begin{align*}{\text{SSIM}}\left( {x,y} \right) &amp; = \frac{{\left( {2{\mu _x}{\mu _y} + {c_1}} \right)\left( {2{\sigma _{xy}} + {c_2}} \right)}}{{\left( {{\mu _x}^2 + {\mu _y}^2 + {c_1}} \right)\left( {{\sigma _x}^2 + {\sigma _y}^2 + {c_2}} \right)}}\end{align*}

where:

${\mu _x}\,and\,{\mu _y}$ are the average of *x* and *y* respectively.

${\sigma _x}^2$ and ${\sigma _y}^2$ are the variance of *x* and *y* respectively.

${\sigma _{xy}}$ is the covariance of *x* and *y*.

${c_1}$ and ${c_2}$ are constants to avoid instability when the denominator is very close to zero.

In this study, we chose MAE, PSNR, and SSIM as evaluation metrics for the following reasons. MAE is particularly useful for understanding the direct differences in intensity values between images. PSNR quantifies the synthesis quality of the predicted images by comparing the maximum possible power of the signal to the power of the noise, making it a widely used metric in image synthesis and denoising tasks. SSIM is designed to be more consistent with human visual perception, making it a valuable metric for evaluating image quality from a perceptual standpoint.

To illustrate the statistical significance of quantitative improvement by the proposed conditional DDPM, paired two-tailed *t*-tests (alpha-0.05) was used for comparison of the outcomes between numerical results groups calculated from 10 patients’ data in the test dataset.

## 3. Results

### 3.1. Image synthetic generation performance comparison

Tables 2 and 3 present the numerical results from the quantitative analysis of synthetic CE-DECT image generation. The results of the paired two-tailed T-tests comparing various image quality metrics between the proposed method and both Pix2PixGAN and CNN for H-CT and L-CT scans are detailed in tables 4 and 5. These tables compare the performance of the proposed C-DDPM method with the two reference models, Pix2PixGAN and CNN, across metrics of MAE, SSIM, and PSNR. The data in tables 2 and 3 demonstrate that the C-DDPM method surpasses both reference models in these metrics. The paired two-tailed t-test provides the significant enhancement achieved by the proposed method over the reference models.

**Table 2.** Comparative analysis of image quality metrics among C-DDPM (proposed method), Pix2PixGAN, and CNN on synthetic H-CT images. The ground truth H-CT images serve as reference for metrics.

	MAE (HU)↓	SSIM↑	PSNR (dB)↑
CNN	58.97 ± 32.28	0.38 ± 0.12	9.39 ± 3.83
Pix2PixGAN	39.35 ± 15.38	0.54 ± 0.24	12.02 ± 2.35
Proposed Method (C-DDPM)	**27.37 ± 3.35**	**0.74 ± 0.22**	**18.51 ± 4.55**

Calculations Exclude Regions Outside Patient Body. Arrow Directions Indicate Superior Performance.

**Table 3.** Comparative analysis of image quality metrics among C-DDPM (proposed method), Pix2PixGAN, and CNN on synthetic L-CT images. The ground truth L-CT images serve as reference for metrics.

	MAE (HU)↓	SSIM↑	PSNR (dB)↑
CNN	50.15 ± 31.28	0.36 ± 0.12	10.48 ± 3.83
Pix2PixGAN	38.27 ± 14.23	0.59 ± 0.24	13.81 ± 2.35
Proposed Method (C-DDPM)	**24.57 ± 3.35**	**0.78 ± 0.22**	**18.91 ± 4.55**

Calculations Exclude Regions Outside Patient Body. Arrow Directions Indicate Superior Performance.

**Table 4.** Paired two-tailed T-test results for image quality metrics (H-CT).

Metric	Comparison	P-value
MAE(HU)	Proposed vs. Pix2PixGAN	<0.01
	Proposed vs. CNN	<0.01
SSIM	Proposed vs. Pix2PixGAN	<0.01
	Proposed vs. CNN	<0.01
PSNR(dB)	Proposed vs. Pix2PixGAN	<0.01
	Proposed vs. CNN	<0.01

**Table 5.** Paired Two-tailed T-test results for image quality metrics (L-CT).

Metric	Comparison	P-value
MAE(HU)	Proposed vs. Pix2PixGAN	<0.01
	Proposed vs. CNN	<0.01
SSIM	Proposed vs. Pix2PixGAN	<0.01
	Proposed vs. CNN	<0.01
PSNR(dB)	Proposed vs. Pix2PixGAN	<0.01
	Proposed vs. CNN	<0.01

**Figure 2.**
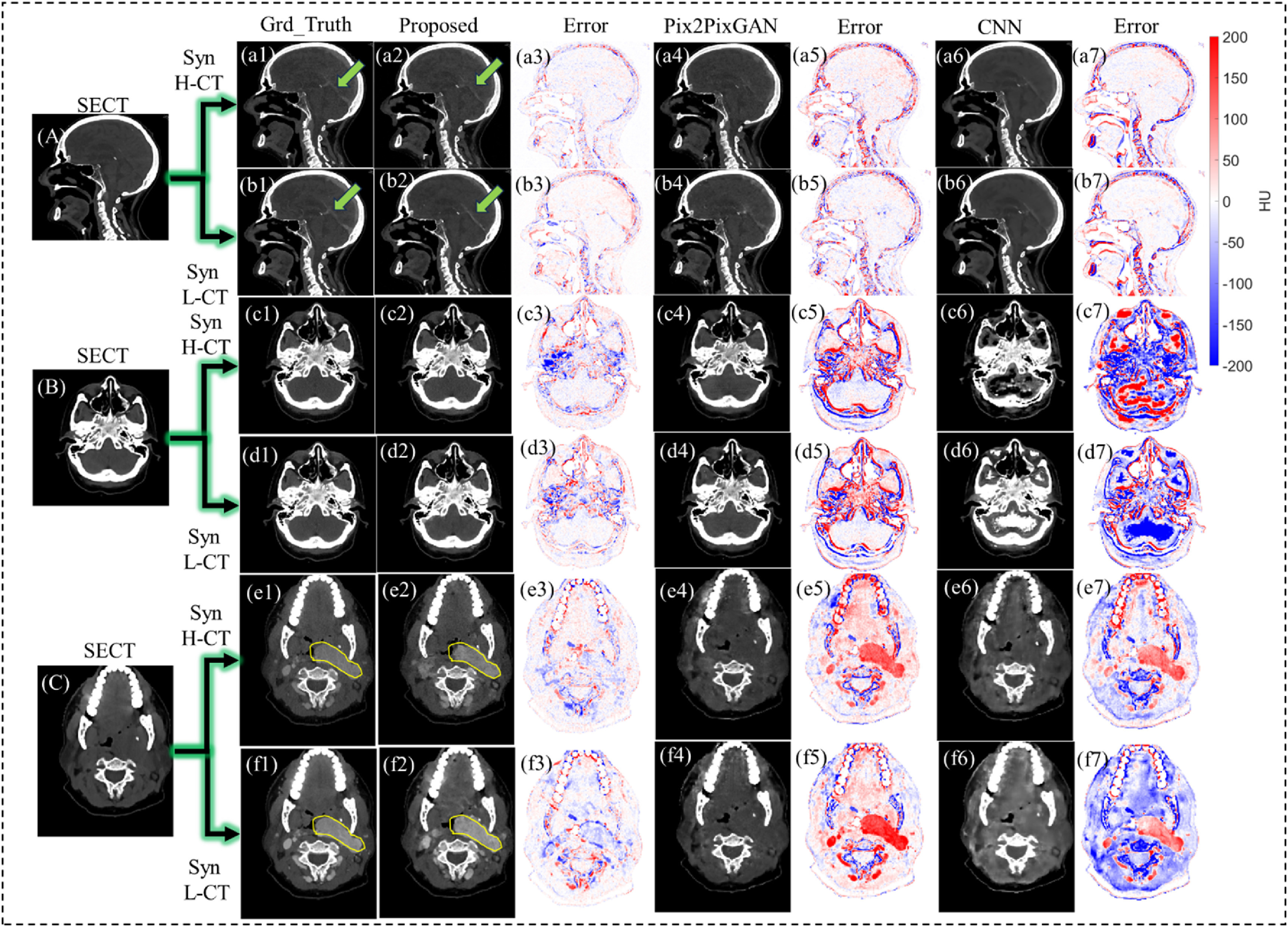
Visual comparison of synthetic CE-DECT images for three patients, generated using the proposed C-DDPM method and reference methods. The figure displays sagittal and axial views of the patients’ SECT in sections (A)–(C). Ground Truth H-CT images are depicted in (a1), (c1), (e1) for the H-CT part and (b1), (d1), (f1) for the L-CT part. Synthetic H-CT and L-CT images created by the Proposed Method (a2), (c2), (e2); (b2), (d2), (f2), Pix2PixGAN (a4), (c4), (e4); (b4), (d4), (f4), and CNN (a6), (c6), (e6); (b6), (d6), (f6) are shown. Additionally, the corresponding error images for the Proposed Method (a3), (c3), (e3); (b3), (d3), (f3), Pix2PixGAN (a5), (c5), (e5); (b5), (d5), (f5), and CNN (a7), (c7), (e7); (b7), (d7), (f7) are also presented, providing a comprehensive visual evaluation. All SECT, synthetic CE-DECT, and ground truth CE-DECT images are presented within a window of −100–500 HU. Error maps are displayed in a window of −200–200 HU. The cerebral artery regions in (a1)–(a7); (b1)–(b7) are specifically indicated by green arrows in (a1), (a2); (b1), (b2). Lesions on axial views (e1)–(e7); (f1)–(f7) are marked with yellow circles in (e1)–(e2); (f1)–(f2) for clear identification.

Figure 2 showcases the synthetic CE-DECT generation capabilities of the proposed method and the reference methods for three patients, in sagittal and axial views. It also illustrates the discrepancies between the synthetic CE-DECT images and the ground truth images. In figure 2, sections (a1)–(a7) and (b1)–(b7) indicate that the reference models perform comparably to the proposed method in this image synthesis task. Each of the three models effectively demonstrates the ability to predict the region of the cerebral artery. However, as depicted in sections (c1)–(f7), the proposed method significantly surpasses the reference models in accuracy. It is noteworthy that all DL models were trained using 2D slices in sagittal views, while the test dataset comprised 2D slices in sagittal, coronal, and axial views. Figure 2 effectively demonstrates that the C-DDPM method excels in generating synthetic CE-DECT images in alternative directions. Figure 2, specifically in sections (e1)–(e7) and (f1)–(f7), illustrates that the proposed method successfully predicts the lesion sites in patients, a task at which the reference models were unable to perform effectively.

**Figure 3.**
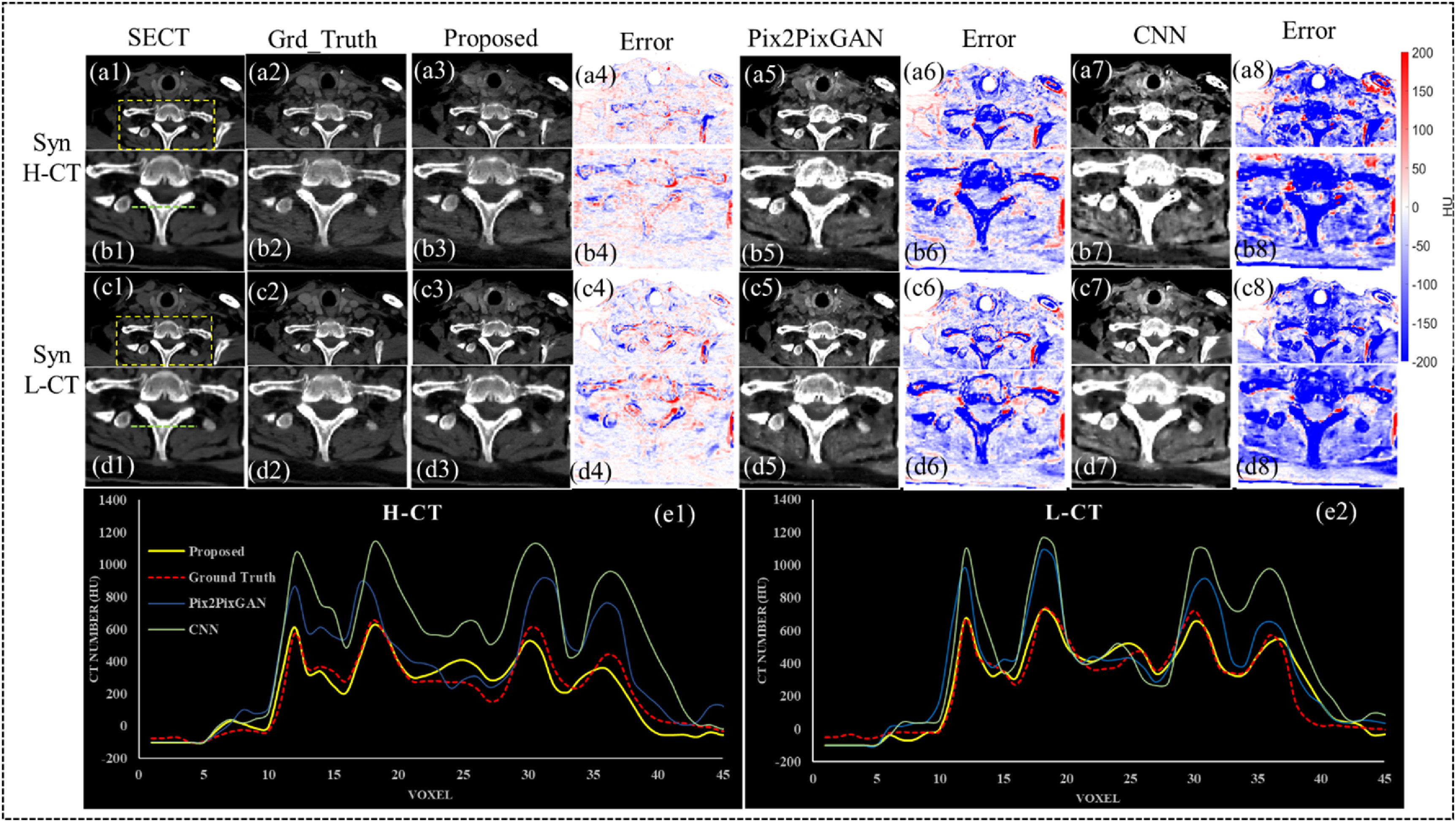
Visual comparison of synthetic CE-DECT images for a single patient and line profile comparison at specific voxels, using the proposed C-DDPM method and reference methods. This figure showcases the axial views of one patient’s SECT (a1), (c1), ground truth H-CT and L-CT images in (a2), (c2), synthetic H-CT and L-CT images generated by proposed method (a3), (c3) Pix2PixGAN (a5), (c5), CNN (a7), (c7), and the Close-ups of specific regions in (a1)–(a8) and (c1)–(c8) are provided in (b1)–(b8) and (d1)–(d8), respectively, highlighted with yellow dashed boxes. Corresponding error maps for the proposed method, Pix2PixGAN, and CNN are displayed in (a4)–(d4), (a6)–(d6), and (a8)–(d8). All SECT, synthetic CE-DECT, and ground truth CE-DECT images are presented within a −100–500 HU window, and error maps in a −200–200 HU window. The line profiles in (e1) and (e2) compare the results across all methods at specific voxels, which are indicated by green dashed lines in (b1) and (d1).

Figure 3 provides a meticulous comparative analysis of synthetic CE-DECT image synthesis using three computational approaches: Pix2PixGAN, a conventional CNN, and the proposed method. The figure is methodically organized into panels to facilitate the visual assessment of the synthesis performance across different models. The initial column of panels (a1), (b1), (c1), (d1) establishes the baseline with axial views of SECT images of a patient. Progressing horizontally, the subsequent panels represent the synthetic H-CT images produced by Pix2PixGAN (a2), (c2), CNN (a5), (c5), and the proposed method (a7), (c7). A parallel layout is adopted for the synthetic Low Energy CT (L-CT) images in panels (b2), (d2), (b5), (d5), and (b7), (d7). Ground truth images for H-CT and L-CT are strategically placed in panels (a6), (c6) and (b6), (d6), respectively, to serve as a gold standard for comparison. Error maps positioned next to each synthetic image series (a3), (c3), (a4), (c4), (a8), (c8), (b3), (d3), (b4), (d4), (b8), (d8) provide a graphical representation of the deviation from the ground truth, quantifying the synthesis accuracy at the pixel level. The performance of the proposed method is notably superior, as evidenced by the comparison of these error maps. At the foundation of figure 3, two-line graphs (e1) and (e2) further dissect the synthesis accuracy by plotting the voxel-wise intensity profiles for both H-CT and L-CT images, with the regions of interest indicated in (b1), (d1). These profiles underscore the precise alignment of the proposed method with the ground truth, in stark contrast to the reference models. This visual juxtaposition not only highlights the proposed method’s superior synthetic image quality but also emphasizes its potential clinical utility, specifically in tasks necessitating exact tissue characterization.

### 3.2. Comparison of carotid artery and lesion site prediction

**Figure 4.**
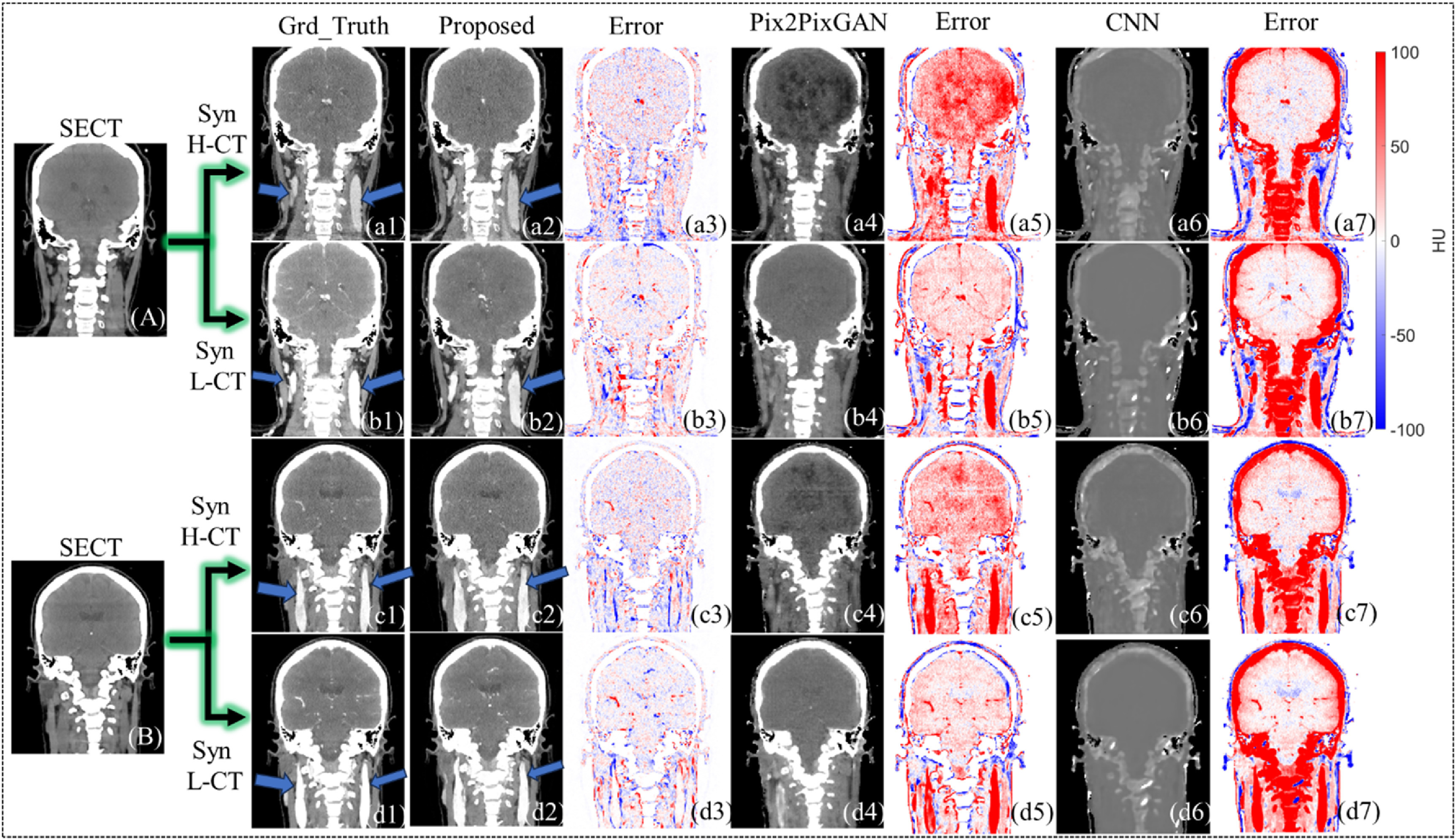
Soft tissue visual comparison of synthetic CE-DECT images for two patients, generated using the proposed C-DDPM method and reference methods. This figure showcases the coronal views of two patients’ SECT in sections (A), (B). Ground truth H-CT and L-CT images are displayed in (a1), (c1) and (b1), (d1) respectively, with synthetic H-CT and L-CT images produced by the proposed method (a2), (c2); (b2), (d2),Pix2PixGAN (a4, c4; b4, d4), CNN (a6), (c6); (b6), (d6). All SECT, synthetic CE-DECT, and ground truth CE-DECT images are presented within a HU window of −100–200, to highlight soft tissue synthesis performance. Corresponding error images for the proposed method, Pix2PixGAN, and CNN are depicted in (a3)–(d3), (a5)–(d5), and (a7)–(d7), within a narrower window of −100–100 HU. The carotid artery (a1)–(d1); (a2)–(d2) are pointed out by blue arrows.

In figure 4, we demonstrate a visual comparison of synthetic CE-DECT image synthesis for two patient cases, categorized into sections (A) and (B). The figure is methodically structured, presenting synthetic H-CT and L-CT images generated by the proposed method, Pix2PixGAN, and CNN, set against the corresponding ground truth images for each case. Adjacent to these images, the error maps are arranged in sequence, providing a clear visual metric of synthesis accuracy. The figure is designed specifically to evaluate the capability of the three methods in delineating the carotid artery, a critical vascular structure. To focus on the accuracy within the soft tissue range, the maximum bone tissue CT number is capped at 200 HU, intentionally omitting the bone tissue discrepancies from the error map analysis. This is evident in the CNN-generated images (a6)–(d6), where a pronounced error in bone tissue representation is observable, contrasting with the Pix2PixGAN and proposed method’s error maps (a5)–(d5), (a3)–(d3), where bone tissue errors are non-existent due to the application’s specific exclusion criteria. In figure 4, panels (a2)–(d2) clearly depict the successful prediction of the carotid artery by the proposed method, demonstrating its efficacy in accurately synthesizing this crucial anatomical feature. In contrast, the reference models, as evidenced in the corresponding panels for Pix2PixGAN and CNN, do not achieve the same level of precision, underscoring the proposed method’s advanced capability in this specific task.

**Figure 5.**
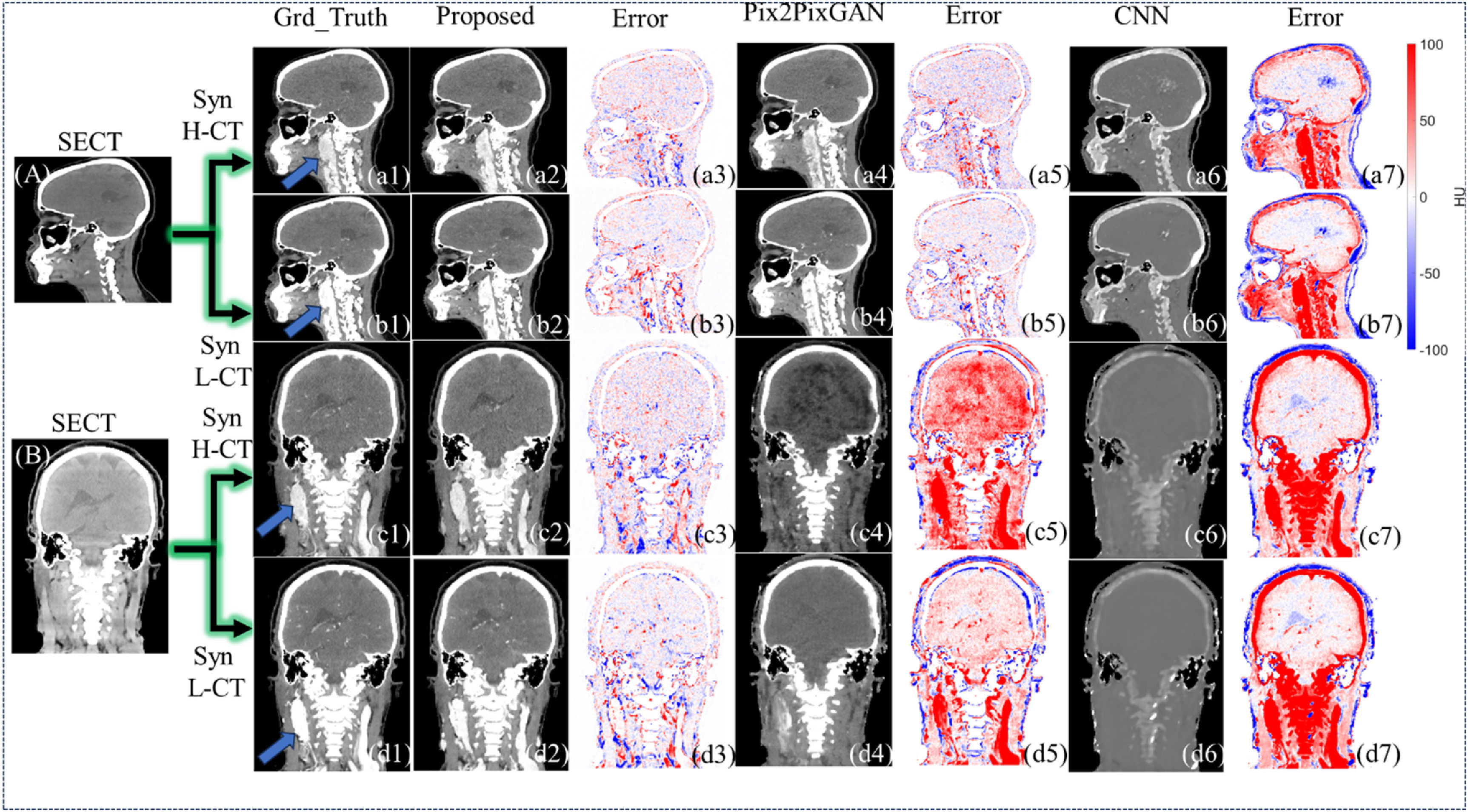
Soft tissue visual comparison and lesion site prediction of synthetic CE-DECT images in one patient, generated using the proposed C-DDPM method and reference methods. This figure showcases the sagittal and coronal views of one patients’ SECT in sections A-B. Ground truth H-CT and L-CT images are displayed in (a1), (c1) and (b1), (d1) respectively, with synthetic H-CT and L-CT images produced by the proposed method (a2), (c2); (b2), (d2), Pix2PixGAN (a4), (c4); (b4), (d4), CNN (a6), (c6); (b6), (d6). All SECT, synthetic CE-DECT, and ground truth CE-DECT images are presented within a HU window of −100–200, to highlight soft tissue synthesis performance. Corresponding error images for the proposed method, Pix2PixGAN, and CNN are depicted in (a3)–(d3), (a5)–(d5), and (a7)–(d7), within a narrower window of −100–100 HU. The lesion site (a1)–(d1) location is pointed out by blue arrows.

Figure 5 presents a detailed visual comparison of synthetic CE-DECT images for a single patient, featuring both sagittal and coronal views. The SECT images are displayed in sections (A) and (B). Synthesized H-CT and L-CT images generated by the proposed method, Pix2PixGAN, and CNN are systematically illustrated in panels (a2), (b2), (c2), (d2), (a4), (b4), (c4), (d4), and (a6), (b6), (c6), (d6), respectively. Error maps, presented alongside each set of synthetic images, quantify the pixel-wise deviation from the ground truth. The CT number values for bone tissue have been deliberately limited to a maximum of 200 HU, focusing the evaluation on the models’ ability to predict lesion sites and delineate the carotid artery. Panels (a1)–(a7) and (b1)–(b7) demonstrate that the reference models yield comparable performance to the proposed method in synthetic CE-DECT image quality. However, panels (c1)–(c7) and (d1)–(d7) reveal a distinguishing feature of the proposed method: it successfully predicts both the lesion site and the carotid artery, a task in which the reference models evidently fall short.

## 4. Discussion

The burgeoning research in medical imaging underscores the pivotal role of CE-DECT images in optimizing treatment planning, particularly for patients undergoing radiation therapy. Leveraging the potential of CE-DECT images, our study introduces a novel framework that capitalizes on a customized C-DDPM to synthesize CE-DECT images from standard non-contrast SECT scans. This paper elucidates the intricacies of the C-DDPM architecture and delineates the tailored hyperparameters that facilitate this transformation. The efficacy of our proposed method is validated through rigorous performance evaluations, where it consistently outperforms established reference methods across key metrics such as MAE, PSNR, and SSIM. These results not only highlight the proficiency of our method in generating high-fidelity synthetic CE-DECT images but also its potential to enhance the precision of radiation therapy planning.

Our work represents a pioneering effort in the application of C-DDPM for the generation of synthetic CE-DECT images from non-contrast SECT images. To the best of our knowledge, this work is the first of its kind in the field. Although there are no published studies for direct comparison between the proposed method, through the following contextual evaluation of related references, it is shown that out approach outperforms to produce high fidelity synthetic images and to predict specific vascular tissues/lesions with superior precision. For instance, Charyyev *et al* employed a 3D residual attention generative adversarial network for the generation of synthetic non-contrast DECT images from non-contrast SECT images (2022). Their model demonstrated a MAE of 36.9 HU for H-CT and 35.8 HU for L-CT. Jeong *et al* utilized a 2D cycleGAN for generating synthetic 50 keV (L-CT) images from 70 keV (H-CT) images, achieving a MSE of 247.90 HU (2023). Liu *et al* implemented a label-GAN for the generation of synthetic non-contrast DECT images from MRI scans. In their approach, the MAE for H-CT was recorded at 80.15 HU, and for L-CT, it was 79.98 HU. Chun *et al* applied a 2D conditional-GAN to generate CE-SECT images from non-contrast SECT images, achieving a MAE of 20.66 HU (2022). While the aforementioned studies by Chun *et al,* Liu *et al* and others vary somewhat from our specific task, their results nevertheless provide valuable reference framework for assessing the effectiveness of our proposed method. Our method achieved a MAE below 30 HU for both H-CT and L-CT, demonstrating its efficacy. Furthermore, our study uniquely illustrates the feasibility of using AI to identify lesion sites and specific vascular structures from non-contrast SECT scans, a capability not evidenced in prior studies. This advancement represents a significant contribution to the field, showcasing the potential of AI in enhancing diagnostic precision in medical imaging, particularly in scenarios where only non-contrast SECT data is available.

The DECT iodine map, a specialized post-processing technique in CE-DECT scans, is renowned for its extensive clinical applications. These applications span various medical fields, such as vascular, cardiac, gastrointestinal, abdominal, and urinary domains (Agostini *et al* 2019). Our proposed method’s capability to produce high-accuracy CE-DECT images enables the generation of a pseudo iodine map from these synthetic CE-DECT images. The proposed method offers significant benefits, particularly for therapy centers not equipped with DECT scanners. Additionally, it has the potential to reduce both the financial costs and the radiation exposure for patients. It is noteworthy that contrast agents injection used in CE-DECT scan can increase organ diagnostic imaging dose by approximately 30% (Mazloumi *et al* 2021). By generating synthetic CE-DECT images, our method could mitigate this increase in radiation exposure, making it a safer and more cost-effective alternative to conventional contrast-enhanced imaging procedures. CE-DECT is recognized for its extensive utility in various medical diagnoses. With our proposed framework providing highly accurate synthetic CE-DECT images, its application can be extended beyond the traditional scope of radiation oncology. Notably, this includes but is not limited to the diagnosis of pulmonary embolism (Grob *et al* 2019), cerebral venous thrombosis (Pontana *et al* 2008), lipid-poor adenoma (Mileto *et al* 2015). This expansion of utility could encompass various other medical specialties, leveraging the detailed and enhanced imaging capabilities of CE-DECT.

In this work, we focus on employing newly developed DL networks to generate synthetic CE-DECT from non-contrast CT scans. We aim to provide insights into both the physical and physiological aspects of this translation task. For physical basis, different tissues in the body exhibit distinct attenuation properties based on their density and atomic number, which influence how x-rays are absorbed or scattered. Non-contrast SECT scans capture detailed anatomical information through these inherent attenuation properties, providing a foundational map that reflects how various tissues will respond to contrast agents. The physiological basis for deriving CE-DECT images from non-contrast SECT scans hinges on the unique compositions and characteristics of different tissues and structures in the body. For instance, vascular structures can be identified based on their baseline attenuation in SECT images and further predicted for contrast enhancement due to their specific physiological characteristics. Those physical and physiological insights enable advance DL models to effectively predict CE-DECT from non-contrast CT scans.

This work reinforces the growing evidence that C-DDPM are superior to GANs and their variants, particularly in tasks involving the synthesis of medical images (Dhariwal and Nichol 2021, Kazerouni *et al* 2022, Müller-Franzes *et al* 2023). A key advantage of C-DDPM lies in their relative ease of training. This factor not only saves time and computational resources but also simplifies the process of hyperparameter tuning. These challenges in GANs can significantly hinder the learning process, but C-DDPM circumvents these pitfalls, leading to a more robust and effective training outcome. Mode collapse, a critical limitation inherent to GANs, can be somewhat mitigated through various strategies (Srivastava *et al* 2017, Thanh-Tung and Tran 2020, Zeng *et al* 2021). However, developing GANs entirely free from mode collapse remains an unresolved challenge in the field. The inherent limitation of GANs, such as the reference method Pix2PixGAN in this work, primarily lies in their tendency towards a lack of diversity in the generated images. This is a crucial factor, especially when the model needs to accurately capture and replicate the complexity and variability of images from different views in CE-DECT. Specifically, if Pix2PixGAN is trained predominantly with images from the sagittal view, its ability to effectively synthesize images from other orientations, such as coronal and axial views, may be significantly compromised. The model might struggle to adapt to the different perspectives and details inherent in these alternate views due to its training on a more homogenous dataset, leading to challenges in achieving accurate and diverse image synthesis across various CE-DECT view orientations. Indeed, this explains why C-DDPM outperformed Pix2PixGAN in this work, particularly in synthesizing images from the two other directional views of CE-DECT—coronal and axial—beyond the sagittal views on which DL models was primarily trained. The performance of CNNs being inferior to that of Pix2PixGAN in generating synthetic CE-DECT images from the two other directional views—apart from the sagittal view. This could be due to CNNs’ typical reliance on labeled data and their potential limitations in capturing the full variability and complexity inherent in different image orientations. Therefore, in the context of synthesizing diverse and complex medical images like CE-DECT, Pix2PixGAN might have a comparative advantage over traditional CNN models, although it still does not match the performance of more advanced models like C-DDPM. While our findings demonstrate the superior performance of the conditional DDPM compared to GAN-based models in our experiments, it would be premature to conclusively state that conditional DDPM outperforms all GAN-based models universally in image synthesis tasks. The efficacy of GAN-based models largely depends on the architecture and optimization of their generator modules. We optimized the generator structure of the reference model by modifying layer configurations and incorporating residual blocks. Although we customized the generator module for optimal performance within the scope of this study, we recognize that our exploration did not cover all possible configurations or optimization pathways. Thus, our results should be interpreted as indicative of the conditional DDPM’s potential rather than a definitive ranking of its capabilities against all GAN-based approaches in image synthesis.

Despite the promising performance of the proposed framework in generating synthetic CE-DECT, there are still limitations in this work. The proposed framework, while demonstrating promising performance in generating synthetic CE-DECT images, does have notable limitations. A primary concern is the significant amount of time required for synthetic image generation using this method, approximately 150 s per slice. This duration is substantially longer than that of the reference methods, which average around 0.15 s per slice. This increased time requirement is mainly due to the intrinsic mechanism of the trained C-DDPM. The C-DDPM, structured to operate with *T* time steps, necessitates the generator’s implementation *T* times for each image generation process. Consequently, the total time taken for sample generation is roughly *T* times longer than that for models based on GANs. This sampling procedure is a significant hindrance in adapting DDPM-based methods for clinical practice, despite their comparable training time to other one-step models like GAN and U-net. The challenge lies in balancing the superior image generation capabilities of C-DDPM with the practical time constraints of medical settings, where swift image processing is often crucial. Addressing this limitation is essential for the wider clinical adoption and practical application of this advanced image synthesis technology. The second limitation of the proposed framework lies in its approach to CE-DECT synthesis, which is treated primarily as an image-to-image translation task. This methodology does not incorporate fundamental physical principles underlying the imaging process. This lack of consideration for the underlying physics could potentially lead to inaccuracies in synthetic images. Therefore, while the image-to-image translation approach effectively generates visually similar images, its utility might be limited in clinical scenarios that demand a deep understanding of the underlying physiological processes. Addressing this gap by integrating physical principles into the model could enhance the accuracy and clinical relevance of the synthetic images generated by the framework. The third limitation of our study pertains to the accuracy of lesion prediction using the proposed framework. In our examination of the predictions across all 10 patients in the test dataset, it was observed that not every lesion could be successfully identified by the proposed method. This limitation is likely attributable to the inherent complexity of lesion sites in HN cancer patients. HN cancers are known for their diverse presentations and the intricate anatomical structures involved, making accurate lesion detection challenging. The training dataset used for our model consisted of data from 120 patients, which, while substantial, might not be comprehensive enough to cover the full spectrum of variability in HN cancers. This limitation in dataset diversity could impede the model’s ability to generalize and accurately predict all lesions across different patient cases. Another limitation is the variability inherent in the outputs generated by the proposed method. Our previous study addressed this issue by averaging several outputs, which helped to reduce the likelihood of illusions produced by DDPM-based models (Chang *et al* 2024). Furthermore, it is important to discuss potential biases in the data and limitations in the model’s applicability to different types of CT scans or patient demographics. The training data used in this study may contain biases related to the specific population or types of CT scans included, which could affect the model’s generalizability. Additionally, the model’s effectiveness may vary across different CT scan settings, such as varying levels of noise, different scanner types, or contrast agent protocols.

• Accelerated Sampling Algorithms: Implementing accelerated sampling algorithms for diffusion models will be crucial in reducing the time required for synthetic image generation. This enhancement is essential for making the method more practical and feasible for clinical applications.
• Incorporation of Fundamental Physics: The integration of fundamental physics principles into the model is a vital step forward. Developing a physics-informed model would ensure that the synthetic images not only visually resemble CE-DECT scans but also accurately represent the physiological and pathological processes. This approach would enhance the clinical relevance of synthetic images.
• Expanding the Training Dataset: To improve the model’s capability in lesion site prediction, particularly in HN cancer patients, it is essential to incorporate a more diverse and extensive dataset. This expansion would allow the model to learn from a broader range of cancer presentations, potentially increasing its accuracy and generalizability. A diverse and representative dataset that encompasses a wide range of patient demographics and scan types can reduce the potential bias of the proposed method.
• Exploring 3D C-DDPM: Currently, the method operates on a 2D slice basis without using patch extraction strategies, primarily due to GPU memory constraints for 3D models. However, exploring a 3D C-DDPM approach could be highly beneficial. Such an approach could yield synthetic images with better structural preservation and a more accurate representation of spatial relationships, leveraging the additional dimensionality.
• Application to Other Anatomical Sites: Given the promising performance of the proposed method, it would be worthwhile to explore its applicability to other anatomical sites. This exploration could reveal the method’s versatility and potential in a wider range of medical imaging applications.

## 5. Conclusion

In this work, we have proposed and validated a DL-based framework for generating synthetic CE-DECT images from non-contrast SECT scans. The results from our quantitative evaluations clearly demonstrate that the proposed method outperforms the reference models, such as Pix2PixGAN and CNN, highlighting the superiority of our proposed C-DDPM in medical image synthesis tasks. The significance of this methodology extends far beyond technical advancement. It is particularly beneficial for patients who are ineligible for iodine contrast scans due to allergies or kidney diseases and for clinical therapy centers that do not have access to DECT scanners. The enhanced stability and performance of our method make it a valuable tool in medical imaging. It holds the potential to significantly improve the accessibility and reliability of CE-DECT, thereby contributing to more accurate and efficient diagnosis and treatment planning in the field of radiation oncology. This approach opens new possibilities in personalized patient care, ensuring that even those who previously could not undergo certain imaging procedures due to medical or equipment limitations can now receive the benefits of advanced diagnostic technologies.

## Data Availability

The data cannot be made publicly available upon publication because the cost of preparing, depositing and hosting the data would be prohibitive within the terms of this research project. The data that support the findings of this study are available upon reasonable request from the authors.
